# Adherence and Compliance With Smoking Cessation in Primary Health Care

**DOI:** 10.7759/cureus.110448

**Published:** 2026-06-08

**Authors:** Alia H Zawawi, Alyah T AlMoneef, Maha AlAssafi, Shaden A Alotaibi, Sarah Basudan

**Affiliations:** 1 Medical Education, King Saud Bin Abdulaziz University for Health Sciences, Ministry of National Guard - Health Affairs, Riyadh, SAU; 2 Family and Community Medicine, King Abdulaziz Medical City, Riyadh, SAU; 3 Family and Community Medicine, King Fahad Medical City, Riyadh, SAU

**Keywords:** barriers to quitting smoking, effects of smoking intervention, smoking cessation, smoking cessation clinics, tobacco dependence, transdermal nicotine patch (tnp), varenicline

## Abstract

Background

Smoking remains a major contributor to preventable disease and mortality. Although smoking cessation programs are widely implemented, quit rates remain low and relapse is common. Pharmacological therapies such as nicotine replacement therapy, varenicline, and bupropion can improve quit rates, but their real-world effectiveness is often limited by poor adherence. This study aimed to evaluate adherence to smoking cessation clinic follow-up, adherence to prescribed pharmacotherapy, smoking cessation outcomes, and associations between selected demographic and treatment-related factors and smoking cessation success.

Methodology

A cross-sectional study was conducted at four primary care smoking cessation clinics affiliated with King Abdulaziz Medical City, Ministry of National Guard Health Affairs, Riyadh, Saudi Arabia. All smokers who attended these clinics were eligible, with pregnant women excluded. Using OpenEpi, a random sample of 344 participants was selected from 1,860 patients. Data were collected from medical records and a validated telephone-administered Arabic questionnaire. Smoking cessation outcomes, pharmacotherapy adherence, and reasons for non-adherence were assessed by participant self-report during telephone interviews. Statistical analysis was performed using SPSS version 26, applying descriptive statistics and chi-square tests with significance set at p-values <0.05.

Results

A total of 344 patients were analyzed, with a mean age of 42 years (SD = ±12); nearly half (49.1%) were aged 40-59 years, and 98.5% were male. Most participants attended only one visit (47.1%). Pharmacologic interventions were prescribed to 44.8% of patients, primarily varenicline (74.7%). The overall smoking cessation success rate was 32.6%. Among participants who reported discontinuation of follow-up visits, lack of motivation was the most commonly reported reason (64.1%). Among participants who provided a reason for non-adherence to pharmacotherapy, medication side effects were the leading reason (24.1%), with nausea being the most frequently reported side effect (9.9%). Younger participants (10-17 years) achieved the highest quit rate (66.7%, p = 0.009); however, this finding should be interpreted cautiously because the age group included only three participants. Clinic visit frequency was not associated with smoking cessation success (p = 0.117). Patients prescribed pharmacotherapy had numerically higher quit rates than those who did not receive pharmacotherapy (35.1% vs. 30.5%); however, this difference was not statistically significant (p = 0.372). Adherence to prescribed medication was significantly associated with higher quit rates (43.4% vs. 14.3%, p = 0.001).

Conclusions

The clinic achieved a quit rate comparable to international standards. Adherence to prescribed pharmacotherapy was associated with higher smoking cessation rates, whereas receipt of pharmacotherapy alone was not significantly associated with smoking cessation success. Low motivation and medication side effects were commonly reported barriers and may represent important targets for interventions aimed at improving engagement and smoking cessation outcomes.

## Introduction

Smoking can lead to many preventable serious diseases, including respiratory and cardiovascular diseases, and it is a well-known contributor to cancer development, such as lung, liver, and colorectal cancer. Thus, it is crucial to keep helping and encouraging smokers to quit [1]. Smoking cessation is arguably the most powerful, cost-effective intervention available in clinical settings for the primary and secondary prevention of smoking-related diseases, disability, and death [2-5]. The ongoing damage and substantial expenses associated with global tobacco addiction highlight the critical importance of prioritizing public health initiatives aimed at smoking cessation [6,7].

In response to the global health hazards linked to smoking, healthcare professionals have been implementing smoking cessation programs and clinics in primary care facilities and hospitals across the country for years [6,8]. However, despite the global implementation of various smoking cessation strategies, cessation rates remain low. Previous evidence indicates that although more than 70% of smokers attempt to quit, only approximately 7% of those who quit without assistance remain abstinent after one year. Furthermore, relapse is particularly common during the first 6-12 months following cessation attempts, with reported relapse rates ranging from 54% to 67% during the first 12 months of abstinence [9-12]. It has been proven that quitting success is higher with assistance than without. Furthermore, effective treatment for tobacco dependence is necessary [13]. The most commonly used pharmacotherapeutic interventions in smoking cessation are nicotine replacement therapy (NRT), varenicline, and bupropion [1]. Participants using smoking cessation medications have been found to be 1.5 to 2 times more likely to quit smoking than those using behavioral therapy alone [14].

However, treatment efficacy as measured in clinical trials does not automatically guarantee effectiveness when used in the real-world setting among the general smoking community. Clinical trial participants tend to receive more attention and more behavioral support than is typically offered to a primary care patient, and trial participants are often healthier and more motivated than their counterparts in the primary care setting. These differences may contribute to significantly lower medication adherence rates and, as a result, to lower rates of smoking cessation in primary care patients. Although 70% of smokers state their intention to quit, fewer than half attempt to quit, and only 4%-7% achieve success in their cessation efforts [15].

Adherence is traditionally defined as “the extent to which a person’s behavior corresponds with agreed recommendations from a health care provider” [16]. One study defined adherence to appointments as having attended at least one follow-up clinic visit within the subsequent 12-month period since the initial visit. The same study defined compliant individuals to medication as those who had at least a 90-day supply during the 113 days following the index prescription fill [15]. One of the primary issues in medical studies and interventions is the high rate of loss to follow-up, which can extend up to 50% [17,18]. Findings show that a significant number of participants do not attend their scheduled follow-up sessions [19-21]. According to research conducted in Turkey, nearly 40.8% of smokers failed to participate in the smoking cessation program after their initial visit [21]. Predictors of non-adherence to appointments included having a low socioeconomic status and having insufficient awareness regarding the health risks [22]. Other potential explanations for non-adherence may be linked to the diminished level of motivation at the time of referral to the intensive approach consultation [12].

According to a study conducted in Minnesota, 22% of patients prescribed smoking cessation medications did not proceed to obtain the prescribed medication [23]. A study regarding the factors linked to smokers’ compliance with prescribed medications highlighted the substantial impact of variables such as the extent of nicotine dependence, withdrawal symptoms, perceptions about medications and quitting, alcohol consumption, stress, depression, social support, and the occurrence of adverse side effects in influencing non-compliance with smoking cessation medications [24]. About 42% of smokers in Saudi Arabia express an intention to quit smoking [25]; however, various obstacles impede their efforts, including low self- efficacy, social pressure, and a lack of awareness about cessation services [26]. Another study conducted in Saudi Arabia observed that non-compliance with smoking cessation medications was notably more prevalent among individuals who had been smoking for an extended period compared to those who had recently started smoking. The impact of friends who smoke was also demonstrated to play a substantial role in non-compliance. Side effects, particularly headaches and difficulty sleeping, were the most significant predictors for discontinuation or non-compliance with prescribed drugs. The study also suggested that intrinsic motivation plays a key role in treatment adherence. Smokers who voluntarily sought smoking cessation support were more likely to remain adherent than those whose attendance was primarily driven by family members or healthcare providers. This explains the importance of personal motivation in ensuring a smoker’s adherence to medication prescriptions for quitting smoking, indicating that external advice may influence the decision to quit, but does not guarantee consistent commitment and successful completion [27].

The primary objective of this study was to evaluate adherence to smoking cessation clinic follow-up and prescribed pharmacotherapy among patients attending smoking cessation clinics in primary healthcare settings. Secondary objectives were to assess smoking cessation outcomes, identify reasons for discontinuation of clinic follow-up and pharmacotherapy, and examine associations between selected demographic and treatment-related factors and smoking cessation success.

## Materials and methods

Study setting

A cross-sectional study was conducted within the smoking cessation clinics located at the four main primary healthcare centers affiliated with King Abdulaziz Medical City (KAMC), Ministry of National Guard Health Affairs (M-NGHA), Riyadh, Saudi Arabia. The four participating centers included the Health Care Specialty Center (HCSC), the National Guard Comprehensive Specialized Clinic (NGCSC), the King Abdulaziz Housing Clinic (Iskan), and the Dirab clinic.

Study subjects

Inclusion criteria comprised all smokers, regardless of age or sex, who attended one of the four participating smoking cessation clinics affiliated with KAMC, M-NGHA, Riyadh, Saudi Arabia. The only exclusion criterion was pregnancy.

Sample size

**Figure 1:**
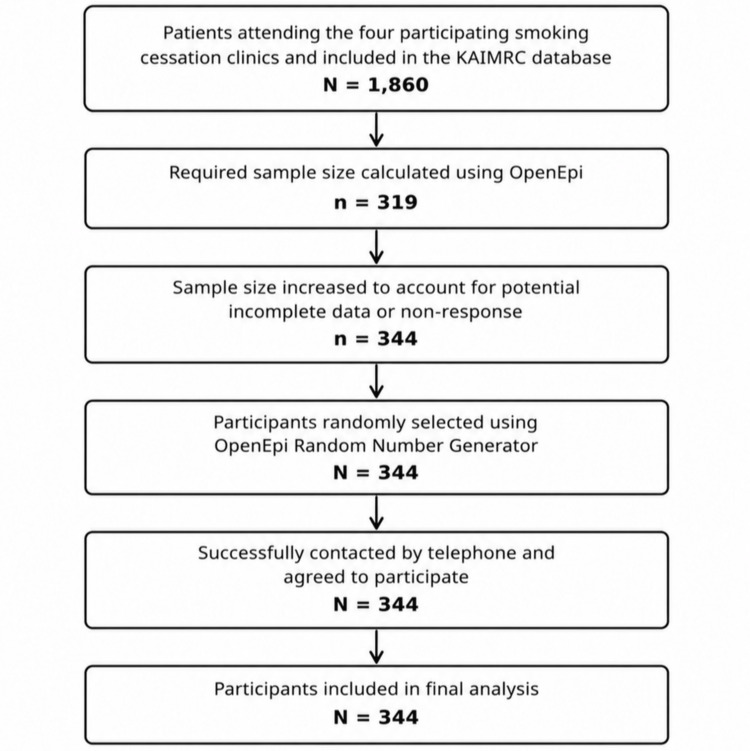
Participant flow diagram. The image illustrates the sample size determination, random sampling process, telephone contact outcomes, and final analyzed sample. No participants were excluded, were unable to be contacted by telephone, or declined participation.

Using the OpenEpi online tool, the sample size was calculated, with a 95% confidence level and a 5% margin of error. Among 1,860 patients who attended smoking cessation clinics, a sample size of 319 participants was required. To account for the possibility of non-response or incomplete data, the calculated sample size was increased to 344 participants. Random sampling was conducted using OpenEpi’s Random Number Generator on the KAIMRC Dataset, which included all smokers, both males and females of various age groups, who attended the smoking cessation clinics in the four participating primary healthcare centers of KAMC, M-NGHA, Riyadh, Saudi Arabia, and were eligible for inclusion. Pregnant women were excluded from the study. Although exclusion of participants aged below 18 years was considered during the initial study planning phase, these participants were retained in the final analysis because they met the eligibility criteria, attended the smoking cessation clinics, and were included in the sampling frame. Therefore, all age groups represented in the clinic population were included in the final dataset. No participants were excluded because of incomplete data, and no records were excluded because of missing data. The participant selection process and final analyzed sample are illustrated in Figure 1.

The Medical Record Numbers of eligible patients were extracted from the KAIMRC database. The following variables were obtained from the BestCare hospital system: demographic data, contact information, visit history, prescriptions for varenicline and/or NRT, and the number of prescriptions.

For the purpose of this study, adherence to smoking cessation clinic attendance was defined as attending at least one follow-up clinic visit within 12 months of the initial smoking cessation clinic visit. Participants who did not attend any follow-up visit during this period were classified as non-adherent. Receipt of pharmacotherapy was determined from medical records and defined as documentation of a prescription for varenicline and/or NRT. Adherence to prescribed pharmacotherapy was assessed through the telephone-administered questionnaire. Participants who reported taking their prescribed smoking cessation medication as directed were classified as adherent, whereas those reporting non-adherence were classified as non-adherent. Smoking cessation was defined as self-reported successful smoking cessation at the time of the telephone interview. Participants who reported having successfully quit smoking were classified as successful quitters.

All 344 selected participants were successfully contacted by telephone in 2025 and agreed to participate in the study. Verbal informed consent was obtained before administration of the questionnaire. The co-investigators introduced themselves and explained the study’s aim. Because participants attended the smoking cessation clinics at different time points, the interval between the initial clinic visit and telephone outcome assessment varied among participants. Smoking cessation outcomes were assessed through participant self-report during the telephone interview. No objective biochemical verification methods were used.

The questionnaire was validated by three consultants with expertise in smoking cessation and primary care to assess the clarity, relevance, and appropriateness of the questionnaire items and was administered in Arabic. The validity of the translation from the original English version was ensured using a forward-backward translation method. The questionnaire assessed smoking cessation outcomes, adherence to clinic follow-up visits, prescribed pharmacotherapy, medication adherence, reasons for discontinuation of follow-up, reasons for non-adherence to pharmacotherapy, and treatment-related side effects. The complete English and Arabic versions of the questionnaire are provided in the Appendices.

Data analysis

Data were analyzed using IBM SPSS Statistics for Windows, version 26.0 (IBM Corp., Armonk, NY, USA). Descriptive statistics (mean, standard deviation, frequencies, and percentages) were used to summarize quantitative and categorical variables. Associations between categorical variables were assessed using the chi-square test. A p-value <0.05 was considered statistically significant, with a 95% confidence interval.

## Results

A total of 344 patients were included in the final analysis. The mean age of the participants was 42 ± 12 years, with a minimum age of 10 years and a maximum age of 84 years. Nearly half (49.1%) of the participants were 40 to below 60 years of age, while 41.3% were aged 18 to below 40 years of age. The sample was predominantly male (98.5%). Based on the predefined definition, 52.9% (182/344) of participants attended at least one follow-up visit within 12 months and were classified as adherent to clinic follow-up. Overall, 47.1% of the participants presented to a single consultation, 38.4% visited two to three sessions, and 14.5% attended more than three times. Further, 44.8% (154/344) of patients were provided with pharmacologic intervention for smoking cessation. Among them, 74.7% (115/154) were prescribed varenicline only, and 39% (60/154) of them received nicotine patches only. Further, 13.6% (21/154) received a combination of both (Table 1).

**Table 1:** Age distribution, clinic attendance, and prescribed pharmacotherapy among study participants. Percentages for age group and number of clinic visits were calculated using the total study population (N = 344). Percentages for pharmacotherapy type were calculated among participants who received pharmacotherapy (N = 154). Because some participants received combination therapy, pharmacotherapy categories are not mutually exclusive, and percentages may exceed 100%.

		Number	Percent
Age	10 to below 18	3	0.9%
	18 to below 40	142	41.3%
	40 to below 60	169	49.1%
	60 and above	30	8.7%
Number of visits	Only one visit	162	47.1%
	Two to three visits	132	38.4%
	More than three visits	50	14.5%
Type of pharmacotherapy	Varenicline	115	74.7%
	Nicotine patches	60	39%
	Combination of both	21	13.6%

The overall smoking cessation success rate was 32.6% (112/344). Among participants who reported discontinuation of follow-up visits, insufficient motivation was the most commonly reported reason (64.1%). Among all participants, 32.9% (113/344) reported adherence to prescribed pharmacotherapy, while 16.3% (56/344) reported non-adherence. The remaining 50.6% (174/344) selected “not applicable,” reflecting that pharmacotherapy had not been prescribed or medication adherence assessment was otherwise not applicable. Among participants who reported non-adherence to pharmacotherapy, experiencing side effects was the most commonly reported reason (24.1%), followed by lack of motivation to quit smoking (20.7%). Nausea was the most frequently reported side effect within this subgroup (9.9%) (Table 2).

**Table 2:** Reasons for discontinuation of clinic follow-up visits and non-adherence to prescribed pharmacotherapy. Percentages were calculated using the total number of responses within each category (N = 259 for reasons for discontinuation of follow-up visits and N = 145 for reasons for non-adherence to pharmacotherapy). The denominator of N = 259 for discontinuation of follow-up visits includes all participants who reported discontinuing clinic attendance and provided a reason, regardless of their classification according to the study definition of clinic follow-up adherence. Similarly, the denominator of N = 145 for reasons for non-adherence to pharmacotherapy reflects all participants who provided a response to this question during the telephone interview and is not restricted to participants classified as non-adherent to pharmacotherapy. Therefore, percentages may not equal the total study population (N = 344). Participants selected one primary reason for each applicable question.

		Number	Percent
Reasons for discontinuation of follow-up visits	Lack of motivation to quit	166	64.1
	Negative experience with previous medication	30	11.6
	Stopped smoking	25	9.7
	Transportation difficulty	19	7.3
	Dissatisfaction with the healthcare provider	15	5.8
	Others	4	1.6
	Total (N)	259	100%
Reasons for non-adherence to the pharmacotherapy	Experienced side effects	35	24.1
	Lack of motivation to quit	30	20.7
	Perceived lack of effectiveness	22	15.2
	The medication was not available	13	9
	Find it difficult to adhere to medication	12	8.3
	Unwilling to use medication to quit	11	7.6
	Lifestyle factors affecting commitment to the medication	10	6.9
	Slow progression	6	4.1
	Others	6	4.1
	Total (N)	145	100%

A statistically significant association was observed between age group and smoking cessation success (p = 0.009). Participants aged 10 to below 18 years demonstrated the highest quit rate of 66.7% compared with the other age groups (Table 3). However, this age group included only three participants; therefore, the estimate may be unstable and should be interpreted with caution, limiting its reliability and generalizability. The frequency of clinic visits was not significantly associated with smoking cessation success (p = 0.117).

**Table 3:** Association between age groups and smoking cessation success.

			Did you successfully quit smoking?		Total (N)	P-value
			Yes	No		
Age	10 to below 18	Count	2	1	3	0.009
		% within age group	66.7%	33.3%	100.0%	
	18 to below 40	Count	58	84	142	
		% within age group	40.8%	59.2%	100.0%	
	40 to below 60	Count	41	128	169	
		% within age group	24.3%	75.7%	100.0%	
	60 and above	Count	11	19	30	
		% within age group	36.7%	63.3%	100.0%	

**Figure 2:**
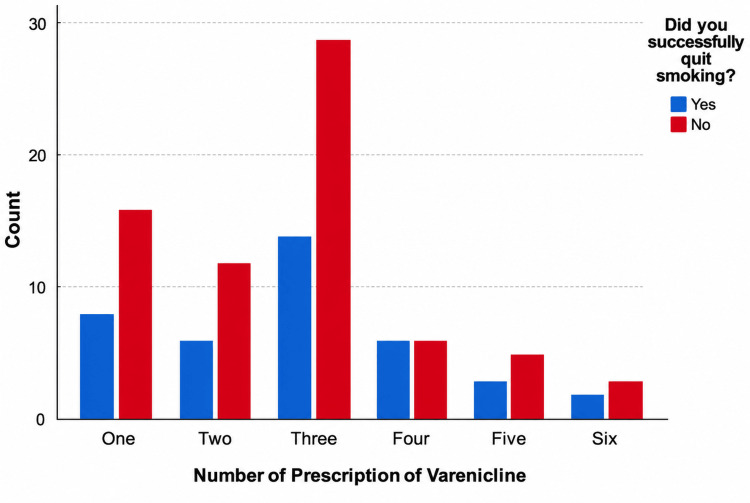
Relationship between the number of varenicline prescriptions and smoking cessation success.

Patients who received pharmacotherapy demonstrated a higher quit rate (35.1%) than those who did not receive pharmacotherapy (30.5%); however, the difference was not statistically significant (p = 0.372). When examining varenicline alone, 35.7% of participants prescribed varenicline successfully quit smoking compared with 31.0% of those who were not prescribed varenicline (p = 0.385). Participants who received repeated varenicline prescriptions had higher smoking cessation rates; however, the difference was not statistically significant (p = 0.649) (Figure 2). Among participants who received NRT, 28.3% achieved smoking cessation compared with 33.5% of those who did not receive NRT (p = 0.442). Participants who received multiple NRT prescriptions had higher smoking cessation rates than those who received a single prescription (50.0% vs. 25.9%); however, the difference was not statistically significant (p = 0.44). Furthermore, 43.4% of participants who adhered to their prescribed pharmacotherapy achieved smoking cessation compared with 14.3% of those who were non-adherent (p = 0.001).

## Discussion

Our smoking cessation clinic achieved a quit rate of 32.6%, which falls within the range reported for outpatient cessation clinics internationally (approximately 21-45%) [28]. This outcome is comparable to findings from other settings; for example, a smoking cessation program in Jazan, Saudi Arabia, reported a 36% success rate [29]. Argüder et al. also found similar outcomes in Turkey, with success noted in around one-third of participants [28]. Such consistency suggests that smoking cessation outcomes observed in our clinics are broadly comparable to those reported by other smoking cessation services.

Interestingly, age appears to influence smoking cessation outcomes, but findings are mixed. In our cohort, younger age was associated with higher cessation success (66.7%), and overall quit success declined with increasing age. However, the highest quit rate was observed in the 10-17-year age group, which included only three participants; therefore, this finding should be interpreted cautiously and considered exploratory, as the small sample size may have produced unstable estimates and limited the generalizability of the observed association. This finding contradicts other studies that reported older adults showing greater success in quitting [30,31]. Our results, by contrast, align with a Turkish report where younger participants exhibited the highest success [28]. These contrasting outcomes indicate that age as a predictor of smoking cessation success is not straightforward and may be modulated by other factors, warranting further investigation.

Nevertheless, early dropout in the program was a significant challenge in our study. Nearly half of the participants (47.1%) attended only a single counseling session, and only 14.5% managed to attend more than three visits. This dropout pattern is consistent with observations from other cessation clinics. Kwon et al. reported that about two-thirds of patients in a Korean smoking cessation clinic came for only one visit [32]. Similarly, a retrospective cohort study from Korea found that more than half of clinic participants dropped out after one or two sessions [33]. This shows that loss to follow-up is a major obstacle for smoking cessation services. However, in our data, the frequency of visits itself was not significantly related to quitting success.

Pharmacotherapy was an important component of treatment in our clinics, although it was underutilized. Only 44.8% of patients received any smoking cessation medication, with varenicline being by far the most commonly prescribed agent (accounting for 74.7% of prescriptions). This observation is consistent with the broader literature indicating that varenicline is one of the most effective first-line treatments for tobacco dependence [34,35], with additional evidence supporting its advantage over bupropion and standard NRT [36]. Adherence to prescribed pharmacotherapy was reported by 32.9% of participants, whereas 16.3% never initiated or prematurely discontinued the medication. Despite the modest overall use of pharmacotherapy, participants who reported adherence to their prescribed medication demonstrated higher smoking cessation rates than those who were non-adherent. However, the association between receipt of pharmacotherapy and smoking cessation success was not statistically significant. Patients who took their medications as directed had a quit rate of 43.4%, compared to only 14.3% among those who were non-adherent. This pronounced difference aligns with prior research demonstrating that adherence to cessation medication is associated with higher success rates [37].

The main reasons patients gave for non-compliance and non-adherence were also informative. Lack of motivation was the most commonly reported reason for discontinuation of follow-up visits (64.1%), and side effects were the leading reason for non-adherence to pharmacotherapy (24.1%). This observation is supported by a study from Jeddah identifying motivation as a critical factor influencing both adherence to clinics and compliance with prescribed medications [38]. These findings suggest that motivation may be associated with adherence and smoking cessation outcomes. Interventions aimed at enhancing patient motivation may help improve clinic attendance and medication adherence, although this relationship warrants further investigation.

Among participants who provided a reason for non-adherence to pharmacotherapy, nausea was the most frequently reported side effect (9.9%). This aligns with previous findings that identify nausea as a common reaction to varenicline [34]. Other side effects noted in our cohort (such as insomnia and vivid dreams) are also well-known reactions to cessation treatments [35]. While these effects are generally mild and transient, they can discourage patients from continuing treatment and should be managed proactively.

Several limitations should be considered. First, as the data were analyzed retrospectively from clinic records and patient self-reports, there is a possibility of reporting bias, recall bias, and missing information. Smoking cessation outcomes were based on self-report without objective biochemical verification, and motivation was assessed using a single questionnaire item rather than a validated instrument. Second, because the follow-up period was relatively short, we focused on initial quit success and did not assess long-term relapse rates. Furthermore, the interval between the initial smoking cessation clinic visit and outcome assessment was not standardized and varied among participants, which may have influenced the reported smoking cessation outcomes. Third, our sample was drawn from a single region and was predominantly composed of male smokers (98.5%), which likely reflects the demographic distribution of smokers attending the participating smoking cessation clinics during the study period. Nevertheless, the limited representation of female smokers may restrict the generalizability of the findings to women and other populations outside the study setting. Fourth, the analysis was limited to univariate comparisons and did not include multivariable logistic regression to identify independent predictors of smoking cessation success while adjusting for potential confounding factors. Consequently, we cannot determine whether medication adherence independently influences smoking cessation success, as the observed association may be affected by unmeasured confounding factors such as motivation or other participant characteristics. Finally, the observational design of the study can only identify associations rather than establish causation; therefore, observed relationships between adherence, compliance, and smoking cessation outcomes should be interpreted with caution.

Despite these limitations, our findings were largely consistent with those of other studies and provide a useful snapshot of outcomes in a real-world smoking cessation clinic setting. Future research using larger multi-center cohorts, standardized follow-up intervals, validated assessment tools, and multivariable analytical approaches is recommended to validate our findings and identify strategies that may improve adherence, compliance, and smoking cessation outcomes.

## Conclusions

In summary, the smoking cessation clinic cohort we studied achieved an encouraging quit rate on par with international benchmarks, while facing significant challenges in patient compliance and medication adherence. Participants prescribed pharmacotherapy had numerically higher smoking cessation rates; however, differences between specific pharmacologic interventions were not statistically significant. Adherence to prescribed pharmacotherapy was associated with significantly higher quit rates. However, given the observational cross-sectional design, the direction and independence of this association cannot be established. The primary obstacles to success were low motivation and treatment side effects, which affected continuous engagement in the program. Addressing these barriers may be associated with improved smoking cessation outcomes and warrants further investigation. Our findings provide useful data for smoking cessation services and may assist healthcare providers and policymakers in strengthening these clinics to support higher quit rates and sustained abstinence.

## Appendices

Appendix 1. Smoking cessation clinic questionnaire (English and Arabic versions)

The following questionnaire was administered by telephone in Arabic to assess smoking cessation outcomes, clinic attendance, medication adherence, reasons for discontinuation, and treatment-related adverse effects.

English

Demographic Information

Age: ______ years

Gender? (Select one answer only):

□ Male

□ Female

Smoking Cessation Outcome

Did you successfully quit smoking?

□ Yes

□ No

Clinic Attendance

If you did not continue attending the smoking cessation clinic, please select the single most important reason? (Select one answer only)

□ Lack of motivation to quit

□ Dissatisfaction with the healthcare provider

□ Negative experience with previous medication

□ Transportation difficulties

□ Other (please specify): __________

Pharmacotherapy

Which smoking cessation medication were you initially prescribed? (Select one answer only)

□ Varenicline

□ Nicotine patches

□ Combination of both

□ None

□ Other (please specify): __________

Were you compliant with the prescribed medication? (Select one answer only)

□ Yes

□ No

If no, please select the single most important reason for discontinuing the prescribed medication? (Select one answer only)

□ Lack of motivation to quit

□ Perceived lack of effectiveness

□ Unwillingness to use smoking cessation medication

□ Slow progress

□ Difficulty adhering to the medication schedule

□ Medication unavailable/out of stock

□ Personal or emotional factors

□ Unclear instructions regarding medication use

□ Lack of understanding of the importance of medication

□ Misinformation regarding medication

□ Feeling stigmatized about medication use

□ Lifestyle factors affecting adherence

□ Side effects (please specify)

Medication Side Effects

-If side effects occurred, please specify one main side effect? (Select one answer only)

□ Nausea

□ Insomnia

□ Disturbed sleep

□ Agitation

□ Drowsiness

□ Constipation

□ Abnormal dreams

□ Headache

□ Skin redness, itching, or burning

□ Intolerance

□ Other (please specify): __________

Arabic:

البيانات الديموغرافية

العمر: ______ سنة

(اختر إجابة واحدة فقط) الجنس-

□ ذكر

□ أنثى

نتيجة الإقلاع عن التدخين

(اختر إجابة واحدة فقط) هل نجحت في الإقلاع عن التدخين؟-

□ نعم

□ لا

متابعة عيادة الإقلاع عن التدخين

(اختر إجابة واحدة فقط) إذا لم تستمر في حضور مواعيد عيادة الإقلاع عن التدخين، فما السبب الرئيسي؟-

□ عدم وجود دافع للإقلاع عن التدخين

□ عدم الرضا عن مقدم الرعاية الصحية

□ تجربة سلبية مع الدواء السابق

□ صعوبات في وسائل النقل

□ سبب آخر (يرجى التحديد): __________

العلاج الدوائي

(اختر إجابة واحدة فقط) ما الدواء الذي وُصف لك في البداية للإقلاع عن التدخين؟-

□ فارينيسلين

□ لصقات النيكوتين

□ مزيج من الاثنين

□ لم يُوصف أي دواء

□ دواء آخر (يرجى التحديد): __________

(اختر إجابة واحدة فقط) هل التزمت باستخدام الدواء الموصوف؟-

□ نعم

□ لا

(اختر إجابة واحدة فقط) إذا كانت الإجابة "لا"، فما السبب الرئيسي للتوقف عن استخدام الدواء؟-

□ عدم وجود دافع للإقلاع عن التدخين

□ الاعتقاد بعدم فعالية الدواء

□ عدم الرغبة في استخدام أدوية للإقلاع عن التدخين

□ بطء التقدم في الإقلاع عن التدخين

□ صعوبة الالتزام بتناول الدواء

□ عدم توفر الدواء

□ عوامل شخصية أو ضغوط نفسية أو عاطفية

□ عدم وضوح تعليمات استخدام الدواء

□ عدم فهم أهمية الدواء

□ معلومات أو معتقدات خاطئة عن الدواء

□ الشعور بالحرج أو الوصمة من استخدام الدواء

□ عوامل متعلقة بنمط الحياة أثرت على الالتزام

□ التعرض لآثار جانبية (يرجى التحديد)

الآثار الجانبية للدواء

(اختر إجابة واحدة فقط) في حال حدوث آثار جانبية، يرجى تحديدها-

□ الغثيان

□ الأرق

□ اضطرابات النوم

□ التهيج أو العصبية

□ النعاس

□ الإمساك

□ أحلام غير طبيعية

□ الصداع

□ احمرار أو حكة أو حرقة في الجلد

□ عدم تحمل الدواء

□ أخرى (يرجى التحديد): __________
